# High Prevalence of Early Parkinson's Disease in Patients With Subtle Parkinsonian Signs

**DOI:** 10.3389/fneur.2021.656679

**Published:** 2021-07-09

**Authors:** Shoichi Sasaki

**Affiliations:** ^1^Department of Neurology, Agano City Hospital, Niigata, Japan; ^2^Department of Neurology, Toyosaka Hospital, Niigata, Japan

**Keywords:** early Parkinson's disease, ^123^I-FP-CIT SPECT, Parkinson's disease, parkinsonism, subtle parkinsonian signs, UPDRS-III, DaTscan images

## Abstract

**Background:** Little is known about how frequently patients with a Unified Parkinson Disease Rating Scale part III (UPDRS-III) score of 3 or 4, including postural and action tremor, could be classified into early Parkinson's disease (PD).

**Objective:** To examine the prevalence of early PD in patients with subtle parkinsonian signs (rest tremor, postural tremor, and rigidity) without bradykinesia, having a UPDRS-III score of 3 or 4.

**Methods:** Parkinsonism was assessed using UPDRS-III based on both the United Kingdom PD Society Brain Bank criteria and the Movement Disorder Society PD criteria. Ninety patients with a UPDRS-III score of 3 or 4, including postural tremor, were evaluated by ^123^I-FP-CIT SPECT (DaTscan), brain MRI, the Mini-Mental State Examination, and smell test. Some patients were additionally examined by ^123^I-metaiodobenzylguanidine myocardial scintigraphy or ^123^I-N-isopropyl-p-iodoamphetamine SPECT.

**Results:** Seventy-five [mean age (standard deviation): 76.9 (8.1)] out of 90 patients (83.3%) showed abnormal findings on DaTscan imaging: 57 out of 75 (76.0%) showed a reduced specific binding ratio (SBR) accompanied by an egg shape pattern (*n* = 37, 49.3%) or a mixed type pattern (*n* = 14, 18.7%), both reduced SBR and increased asymmetry index (AI) with a normal shape (*n* = 4, 5.3%), and reduced SBR only (*n* = 2, 2.7%); 18 (24.0%) showed an egg shape pattern or a mixed type pattern without reduced SBR. In other words, 69 out of 75 patients (92.0%) showed either an egg shape or a mixed type pattern with or without reduced SBR. All patients were free of dementia, and their olfactory function was significantly impaired compared with controls (*n* = 141) on the odor-stick identification test for Japanese (*p* < 0.0001).

**Conclusions:** The prevalence of patients with subtle parkinsonian signs having a UPDRS-III score of 3 or 4, including postural tremor, is unexpectedly high in daily clinical practice, and most of these patients could be categorized into mild early-stage PD.

## Introduction

According to both the United Kingdom Parkinson disease (UKPD) Society Brain Bank criteria (1) and the Movement Disorder Society (MDS) clinical diagnostic criteria for Parkinson's disease (PD) (MDS-PD criteria) (2), the presence of parkinsonism is defined as bradykinesia, in combination with either rest tremor, rigidity, or both, and to make a diagnosis of a manifest parkinsonism, the Unified Parkinson Disease Rating Scale part III (UPDRS-III) score (3) should be at least 5 after removing tremor (1, 2). Moreover, possible subthreshold parkinsonism on expert examination is defined as a UPDRS-III score >3, excluding action tremor (4, 5).

The author noticed that subtle parkinsonian signs such as rest tremor, postural tremor, and rigidity without bradykinesia were unexpectedly prevalent in outpatients in daily clinical practice. The author performed a cross-sectional study of how frequently patients with subtle parkinsonian signs having a UPDRS-III score of 3 or 4, including postural tremor, could be classified into early-stage PD, using ^123^iodine-labeled N-(3-fluoropropyl)-2β-carbomethoxy-3β-(4-iodophenyl) nortropane (^123^I-FP-CIT) single photon emission computed tomography (SPECT) (DaTscan), brain MRI, the Mini-Mental State Examination (MMSE), and smell test. Some patients additionally had ^123^I-metaiodobenzylguanidine (MIBG) myocardial scintigraphy or ^123^I-N-isopropyl-p-iodoamphetamine (^123^I-IMP) SPECT.

## Materials and Methods

### Control Individuals

As controls, a total of 141 individuals with a UPDRS-III score of 0 between 60 and 92 years of age were recruited from non-neurological outpatients such as controlled hypertensive patients from Agano City Hospital, Toyosaka Hospital, and Otsuki Municipal Central Hospital in Japan, along with medical staff and administrative assistants there, and other community-dwelling individuals as well. All control individuals had no cognitive impairment [defined as MMSE score ≥25 in individuals of 65 years and under, and ≥24 in those of over 65 years (6, 7), taking into account educational attainment].

### Patients

A total of 90 patients with subtle parkinsonian motor signs such as rest tremor, postural tremor, and rigidity without bradykinesia, having a total of UPDRS-III score of 3 or 4 from Agano City Hospital, Toyosaka Hospital, and Otsuki Municipal Central Hospital in Japan, were enrolled. Patients with hypokinesia or rigidity by arthritis and those with stooped posture by osteoporotic kyphosis were excluded. Patients with other forms of parkinsonism such as atypical neurodegenerative parkinsonism [corticobasal syndrome (CBS), multiple system atrophy with predominant parkinsonism (MSA-P), and progressive supranuclear palsy (PSP)], vascular parkinsonism (lower body parkinsonism with predominant gait impairment), and drug-induced parkinsonism were clinically ruled out.

Among various ancillary diagnostic tests, the author put emphasis on DaTscan imaging and a smell test to detect mild early PD based on the most predictive tests as published by the MDS (8, 9). In early stages of PD, the overall accuracy of DaTscan imaging in diagnosing early PD is very high (10, 11). The images were evaluated by visual assessment of tracer binding (12) and by quantitative analysis calculating the values of the left and right specific binding ratio (SBR) and asymmetry index (AI) (13). The SBR was estimated using a volume of interest (VOI) technique that accounts for the partial volume effect by deriving the “specific” count concentration in the striatum from a measure of the total counts (13). The whole brain accumulation concentration was used as a reference (14). The result of DaTscan imaging was defined “abnormal” when a reduced SBR (13), an abnormally increased AI (13), or abnormal patterns such as an egg shape (bilateral reduction of tracer uptake in the putamen and normal or almost normal uptake in caudate nuclei) (grade 2) and a mixed type (normal or almost normal tracer uptake in bilateral caudate nuclei with asymmetrical tracer uptake reduction in the putamen of one side) (grade 3) (12) were recognized by visual assessment. The SBR reduction and an egg shape pattern are regarded as high evidence of PD (12, 14), which makes a PD diagnosis more probable or certain. On the other hand, MIBG myocardial scintigraphy for the diagnosis of PD is highly valuable in advanced patients with a PD of Hoehn-Yahr stage III or more, but in the early stages of PD (Hoehn-Yahr stage 1 and 2), it is less valuable in diagnosis due to its comparatively reduced sensitivity (15), although there are some reports that MIBG myocardial scintigraphy is highly effective for detecting early PD and for distinguishing PD from other parkinsonism, specifically MSA-P, PSP, and CBS (16). In this study, all patients with parkinsonian signs were examined by DaTscan imaging, and MIBG myocardial scintigraphy was additionally performed in a part of patients who subjectively complained of slowness of movement to differentiate idiopathic PD from atypical parkinsonism such as MSA-P, PSP, and CBS characterized by akinetic/rigid-dominant parkinsonism and rapid clinical deterioration.

A smell test was performed in all patients, using an odor-stick identification test for Japanese (OSIT-J; Daiichi Yakuhin Sangyo, Tokyo) with 12 daily odorants familiar to Japanese individuals. The OSIT-J score was counted in three endpoints: the numbers of correct answers, responses of *indistinguishable*, and responses of *odorless*. Comorbid medical conditions, such as chronic rhinitis and current heavy smoking (>20 cigarettes per day), were excluded. Moreover, since it has been reported that MCI or mild AD and parkinsonism have synergistic effects on olfactory dysfunction (17), cognitive dysfunction was ruled out in this study.

Patients with amnestic cognitive impairment were ruled out by MMSE: cognitive impairment was defined as an MMSE score ≤24 in patients of 65 years and under, and ≤23 in patients of over 65 years (6, 7), taking into consideration their educational attainment.

Brain MRI (1.5 T) was performed in all patients to exclude vascular parkinsonism (white matter lesions, lacunes in basal ganglia, and cerebral microbleeds), cognitive impairment due to cerebral infarction and small vascular diseases caused by cerebral organic changes, and neuroimaging signs typical of CBS, MSA-P, or PSP. Brain MRI images were also analyzed to evaluate the degree of atrophy of medial temporal structures (the entorhinal cortex, hippocampus, and amygdala), determined as a target volume of interest (VOI), using voxel-based specific regional analysis system for Alzheimer's disease (VSRAD) software to detect very mild Alzheimer's disease (AD) (18). The degree of medial temporal atrophy was calculated using the averaged VSRAD *Z*-score on the target VOI (0–1: no atrophy; 1–2: mild; 2–3: moderate; >3: severe) (VSRAD advance 2, Eisai Co, Tokyo, Japan). ^123^I-IMP SPECT (three-dimensional stereotactic surface projection: 3D-SSP) was additionally used to patients who subjectively complained of cognitive decline, because ^123^I-IMP SPECT (3D-SSP) is more sensitive compared to brain MRI in case there are subjective cognitive deficits that would merit performing the I-IMP in addition to the MRI (19).

As for association between a UPDRS-III score and parkinsonism, parkinsonism is defined as a total score of UPDRS-III of at least 5 after removing tremor, according to the UKPD Society Brain Bank criteria (1) and MDS-PD criteria (2). Possible subthreshold parkinsonism on expert examination is defined as a UPDRS-III score >3, excluding action tremor (4, 5). Moreover, the total score on UPDRS-III reaches an abnormal level at an estimated 4.5 years prior to clinical diagnosis of parkinsonism: a UPDRS score >4 identified prodromal parkinsonism with 88% sensitivity and 94% specificity 2 years before diagnosis; removal of action tremor scores improved the sensitivity to 94% and specificity to 97% at 2 years before diagnosis (cutoff > 3) (5). In this study, the author defined mild early PD as PD showing rest tremor with or without postural tremor, and rigidity according to both UKPD Brain Bank criteria (1) and MDS-PD criteria (2) and having a UPDRS-III score of 3 or 4 with a positive DaTscan such as a reduced SBR or abnormal patterns.

### Statistical Analysis

The demographic and clinical features (age, sex, UPDRS-III score, disease duration, MMSE, educational attainment, and VSRAD *Z*-score) of the control subjects and the patients are shown in Table 1. The data were shown as mean (standard deviation: SD). The difference by sex between patients with a positive DaTscan and those with a negative DaTscan was analyzed using Fisher's exact test, which is suitable for comparison between two groups. The other items (age, UPDRS-III score, disease duration, MMSE score, years of schooling, and VSRAD *Z*-score) were compared among three groups, i.e., controls and two patient groups with a positive or negative DaTscan, and so were assessed by analysis of variance (ANOVA). Disease duration was defined as elapsed months after the patients had first noticed motor symptoms or since diagnosis had been confirmed by the doctor, if the patients did not recognize any parkinsonian signs.

**Table 1:** Demographic and clinical characteristics of controls and patients with a UPDRS score of 3 or 4.

**Demographic and clinical characteristics**	**Controls, *n* = 141**	**Patients with a positive DaTscan, *n* = 75**	**Patients with a negative DaTscan, *n* = 15**	Patients with a positive DaTscan vs. patients with a negative DaTscan, *p*-value^*^
Age, y, mean ± SD (range)	75.0 ± 8.7 (60–92)	76.9 ± 8.1 (55–92)	75.5 ± 7.7 (62–88)	0.536
Sex, female, *n* (%)	87 (61.7%)	45 (60.0%)	7 (46.7%)	0.251
UPDRS part III score, mean ± SD		3.8 ± 0.4	3.8 ± 0.4	0.905
Disease duration, mo, mean ± SD (range)		10.9 ± 9.6 (1–36) (*n* = 35)	10.1 ± 9.5 (0.5–24) (*n* = 5)	0.864
MMSE score, mean ± SD (range)	29.0 ± 1.1 (26–30)	27.8 ± 2.2 (26–30)	27.5 ± 2.7 (24–30)	0.666
Years of schooling, mean ± SD (range)	10.7 ± 2.5 (8–16)	10.0 ± 2.5 (6–16)	10.3 ± 2.3 (8–16)	0.699
VSRAD Z-score, mean ± SD (*n*)		0.997 ± 0.52 (*n* = 73)	0.937 ± 0.42 (*n* = 13)	0.695

*Data are presented as mean ± standard deviation (SD)*.

* *p-values were assessed by Fisher exact test (sex) or analysis of variance (ANOVA) (others)*.

*DaTscan, ^123^iodine-labeled N-(3-fluoropropyl)-2β-carbomethoxy-3β-(4-iodophenyl) nortropane single photon emission computed tomography; MMSE, Mini-Mental State Examination; UPDRS, Unified Parkinson's Disease Rating Scale; VSRAD, Voxel-based specific regional analysis system for Alzheimer's disease*.

In the first comparison of the smell test, the three endpoints (correct answers, responses of *indistinguishable*, responses of *odorless*) were compared between the control subjects and the combined patient group (patients with a positive DaTscan and those with a negative DaTscan), using a model that includes age and sex as a covariance [analysis of covariance (ANCOVA)]. In the second comparison, the three endpoints in patients with a positive DaTscan were compared with those in patients with a negative DaTscan only if the first comparison (controls vs. combined patient group) showed statistically significant differences after adjustment of multiplicity by Holm's method. The multiplicity of the three endpoints was adjusted by the Bonferroni method. The data analysis was conducted with SAS statistical software, version 9.4 (SAS Institute, Cary, NC). The author considered two-sided *p* < 0.05 to be statistically significant.

## Results

### Control Individuals

A total of 141 control individuals were investigated. Their demographic and clinical characteristics are presented in Table 1. The mean age (SD) was 75.0 (8.7) years, and 87 (61.7%) were women. The mean age (SD) of the men was 73.7 (8.7) and that of women was 75.7 (8.7), revealing no significant difference (ANOVA, *p* = 0.173). The mean MMSE score (SD) and the mean educational attainment (SD) was 29.0 (1.1) and 10.7 (2.5) years, respectively. The mean scores (SD) of the three endpoints (correct answers, responses of *indistinguishable*, and responses of *odorless*) on the OSIT-J were 7.3 (1.8), 0.6 (1.1), and 0.2 (0.5), respectively (Table 2).

**Table 2:** Comparison of the smell test results between controls and the combined patient group and between the patient groups.

**OSIT-J score**	**Controls, *n* = 141**	**Patients with a positive DaTscan, *n* = 75**	**Patients with a negative DaTscan, *n* = 15**	**Controls vs. combined patient group, *n* = 90, *p*-value^a^**	**Patients with a positive DaTscan vs. patients with a negative DaTscan *p*-value^b^**
Correct answers	7.3 ± 1.8	4.2 ± 2.2	5.8 ±1.4	<0.0001^*^	0.008^*^
Responses of *indistinguishable*	0.6 ± 1.1	1.9 ± 2.1	0.5 ± 1.1	<0.0001^*^	0.016^*^
Responses of *odorless*	0.2 ± 0.5	1.4 ± 2.4	0.5 ± 0.9	<0.0001^*^	0.182

*Data are presented as mean ± standard deviation*.

a *Analysis of covariance (ANCOVA) (covariates: age, sex)*.

b *ANCOVA (covariates: age, sex, UPDRS part III, disease duration, Mini-Mental State Examination, education, voxel-based specific regional analysis system for Alzheimer's disease); DaTscan, ^123^iodine-labeled N-(3-fluoropropyl)-2β-carbomethoxy-3β-(4-iodophenyl) nortropane single photon emission computed tomography; OSIT-J, the odor-stick identification test for Japanese*.

### Patients

A total of 90 patients with subtle parkinsonian signs such as rest tremor, postural tremor, and rigidity without bradykinesia, having a total of UPDRS-III score of 3 or 4, were studied. Their demographic and clinical characteristics are presented in Table 1. Eighty-eight patients showed rest tremor, postural tremor, and rigidity (a UPDRS-III score of 3 or 4), whereas two patients exhibited rest tremor and rigidity (a UPDRS-III score of 3). Among them, eight patients subjectively complained of slowness of movement, but objectively none of them demonstrated bradykinesia with a neurological examination (UPDRS-III motor scale). All the patients showed no hindrance to activities of daily life. Three male patients (58, 67, and 72 years) had rapid eye movement sleep behavior disorder (RBD) diagnosed by RBDSQ-J (20).

### Patients With a UPDRS-III Score of 3 or 4 With a Positive DaTscan

Of 90 patients, 75 (83.3%) showed abnormal findings on DaTscan imaging. The demographic and clinical characteristics of these patients with a UPDRS-III score of 3 (*n* = 25) or 4 (*n* = 50) are presented in Table 1. The mean age (SD) of 75 patients was 76.9 (8.1), and 45 (60.0%) were women, showing no significant difference compared with the controls (ANOVA, *p* = 0.103, Fisher's exact test, *p* = 0.460, respectively). The mean age (SD) of the men was 75.5 (8.5) and that of women was 77.9 (7.8), indicating no significant difference between the sexes (ANOVA, *p* = 0.209). Forty out of 75 patients (53.3%) did not recognize parkinsonian symptoms, ascribing their parkinsonian signs to the aging process instead. Thirty-five patients complained of their parkinsonian symptoms: 28 patients noticed rest tremor, while seven patients subjectively complained of slowness of movement. The mean disease duration (SD) (*n* = 35) was 10.9 (9.6) months. The mean MMSE score (SD) (*n* = 75) was 27.8 (2.2). The mean educational attainment (SD) was 10.0 (2.5) years. The mean VSRAD *Z*-score (SD) in the 73 patients was 0.997 (0.52).

**Figure 1:**
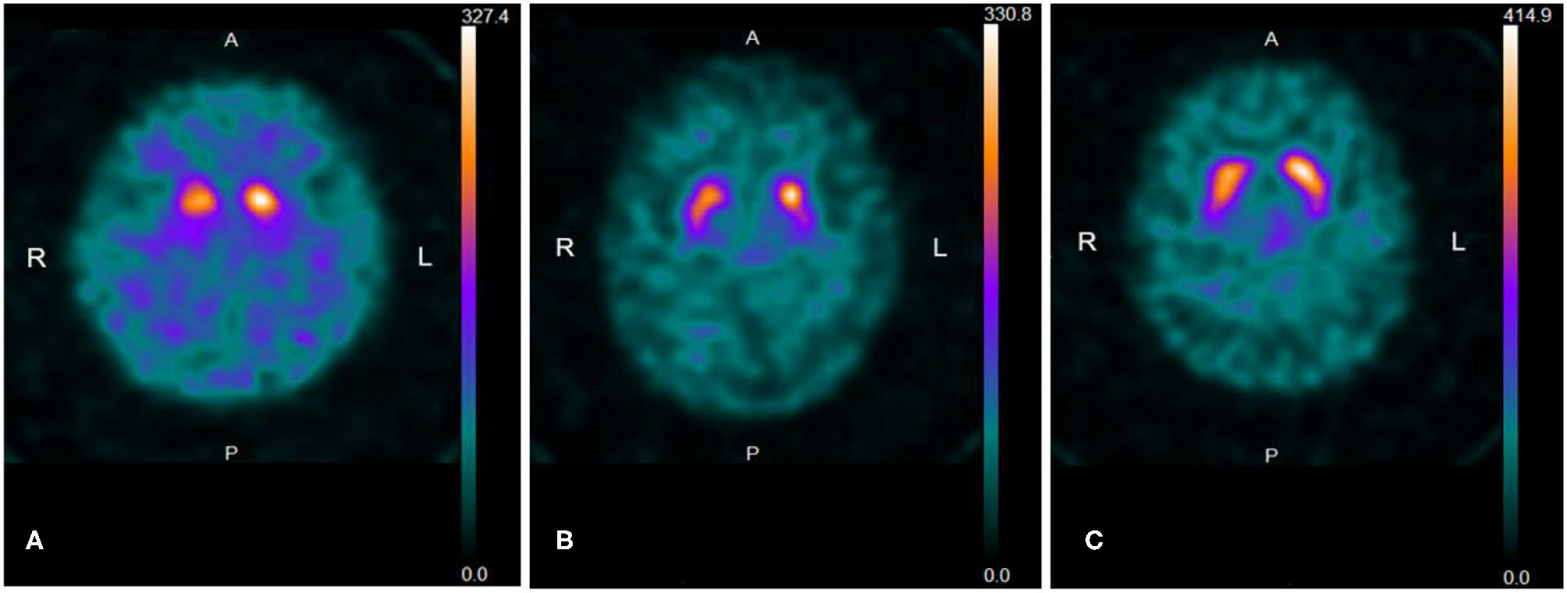
**(A**) Representative ^123^I-FP-CIT SPECT (DaTscan) image of an “egg shape” pattern. The patient is a 78-year-old woman with a UPDRS-III score of 4. Uptake of DaTscan is almost absent in both putamina and normal or almost normal in caudate nuclei, showing an “egg shape” pattern. SBR is bilaterally reduced (R: 1.61; L: 1.96, Avg; 1.79) and AI is higher (19.6%) than the normal range (<10.9%). SBR: specific binding ratio, AI: asymmetry index. **(B)** Representative DaTscan image of a “mixed type” pattern. The patient is an 85-year-old man with a UPDRS-III score of 4. A DaTscan image shows normal or almost normal tracer uptake in bilateral caudate nuclei with asymmetrical tracer uptake reduction in the putamen of the left side by visual assessment, showing a “mixed type” pattern. SBR is bilaterally reduced (R: 2.60; L: 2.95, Avg: 2.77) and AI is increased (12.7%) (normal range: <11.05%). **(C)** The patient is an 80-year-old woman with a UPDRS-III score of 4. The pattern of uptake of DaTscan is almost normal by visual assessment. However, SBR is reduced in the striata with right-side predominance (R: 3.09; L: 3.83, Avg: 3.46), and AI is increased (21.3%) (normal range: <11.05%).

Fifty-seven out of 75 patients (76.0%) showed a reduced SBR accompanied by an egg shape pattern (grade 2) (*n* = 37, 49.3%) (Figure 1A) or a mixed type pattern (grade 3) (*n* = 14, 18.7%) (Figure 1B), both reduced SBR and increased AI with a normal shape (*n* = 4, 5.3%) (Figure 1C), and reduced SBR only (*n* = 2, 2.7%); 18 (24.0%) showed an egg shape pattern (*n* = 8) or a mixed type pattern (*n* = 10) without reduced SBR. In other words, by visual assessment, 69 out of 75 patients (92.0%) showed either an egg shape or a mixed type pattern with or without reduced SBR. The degree of DaTscan SPECT abnormalities did not necessarily correlate with a UPDRS-III score. In addition, all the five patients who subjectively complained of slowness of movement and underwent additional MIBG myocardial scintigraphy showed low MIBG uptake as well as a reduced SBR on DaTscan imaging, although they did not show the objective evidence. A total of 19 patients with subjective cognitive decline [mean MMSE score (SD): 27.4 (2.5)] who underwent ^123^I-IMP SPECT (3D-SSP) showed no significantly reduced blood flow, particularly in the parietal lobe, posterior cingulate gyrus, and precuneus.

The mean scores (SD) of correct answers, responses of *indistinguishable*, and responses of *odorless* on the OSIT-J in 73 patients were 4.2 (2.2), 1.9 (2.1), and 1.4 (2.4), respectively (Table 2). Comparisons of the three endpoints between the controls and the patients showed statistically significant differences in every endpoint (ANCOVA, setting age and sex as covariates, *p* < 0.0001).

### Patients With a UPDRS-III Score of 3 or 4 With a Negative DaTscan

**Figure 2:**
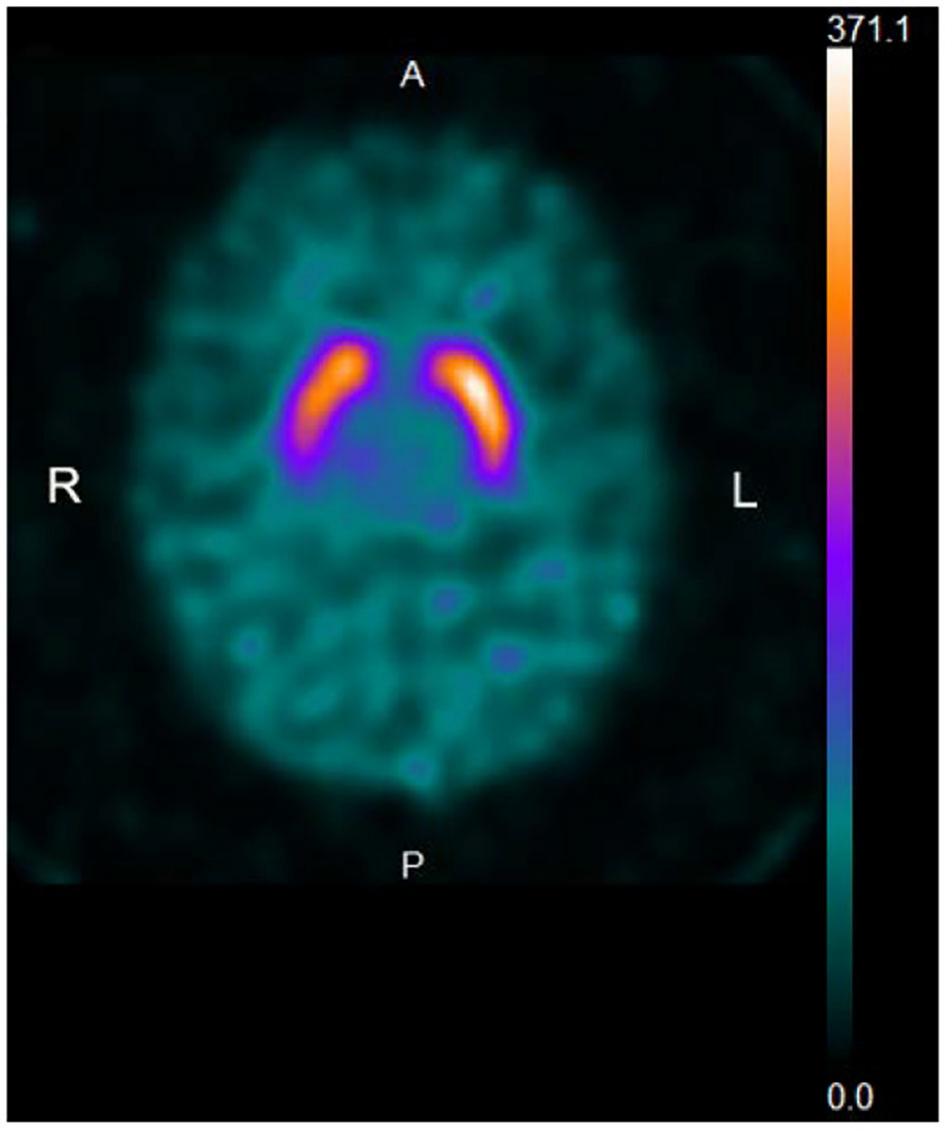
Representative DaTscan image of a normal pattern. The patient is an 84-year-old woman with a UPDRS-III score of 4. A DaTscan image shows a normal pattern by visual assessment, and normal values of SBR (R: 6.04, L: 6.67, Avg: 6.35) and AI (10.0%) (normal range: <11.05%).

A total of 15 patients (16.7%) with a UPDRS-III score of 3 (*n* = 4) or 4 (*n* = 11) did not reveal dopamine deficiency by DaTscan imaging (Figure 2). The demographic and clinical characteristics are demonstrated in Table 1. The mean age (SD) of 15 patients was 75.5 (7.7), and 7 (46.7%) were women. The mean age (SD) of the men was 72.9 (9.0) and that of the women was 78.6 (5.0), showing no significant difference (ANOVA, *p* = 0.161). Four patients noticed rest tremor, whereas one patient subjectively complained of slowness of movement. Ten out of 15 patients did not recognize parkinsonian symptoms. The mean disease duration (months) (SD) (*n* = 5) was 10.1 (9.5). The mean MMSE score (SD) (*n* = 15) was 27.5 (2.7). The mean educational attainment (SD) was 10.3 (2.3) years. The mean VSRAD *Z*-score (SD) (*n* = 13) was 0.937 (0.42). One patient with subjective cognitive decline (MMSE: 30) who underwent ^123^I-IMP SPECT (3D-SSP) showed no significantly reduced blood flow.

The mean scores (SD) of correct answers, responses of *indistinguishable*, and responses of *odorless* on the OSIT-J in 15 patients were 5.8 (1.4), 0.5 (1.1), and 0.5 (0.9), respectively (Table 2), showing statistically significant differences in correct answers (*p* = 0,002) and responses of *odorless* (*p* = 0.022) compared to controls (ANCOVA, setting age and sex as covariates), although no significant difference was shown in the responses of *indistinguishable* (*p* = 0.811).

### Comparison of the Smell Test Results Between Controls and the Combined Patient Group

Comparisons of the smell test results were performed in the three endpoints between controls and the combined patient group (patients with a positive DaTscan and those with a negative DaTscan) [correct answers, 4.5 (2.2); responses of *indistinguishable*, 1.7 (2.0); responses of *odorless*, 1.2 (2.3)] by ANCOVA, setting age and sex as covariates. Significant differences were statistically demonstrated between the two in every endpoint (*p* < 0.0001) (Table 2).

### Comparison of the Smell Test Results Between the Patient Groups

Comparisons of the smell test results were made in the three endpoints by ANCOVA between patients with a positive DaTscan and those with a negative DaTscan, setting age, sex, MMSE, educational attainment, disease duration, UPDRS-III, and VSRAD as covariates. Patients with DaTscan abnormalities had significantly less correct answers compared to those without DaTscan abnormalities [4.2 (2.2) vs. 5.8 (1.4), *p* = 0.008] and significantly more responses of *indistinguishable* [1.9 (2.1) vs. 0.5 (1.1), *p* = 0.016] (Table 2). No significant difference was observed in the score of responses of *odorless* (*p* = 0.182) (Table 2).

## Discussion

In this study, the prevalence of patients with subtle parkinsonian signs (rest tremor, postural tremor, and rigidity) without bradykinesia, having a total UPDRS-III score of 3 or 4 (a total UPDRS-III score of 2 or 3, excluding postural tremor), was unexpectedly high in the neurological outpatient setting, and these patients, for the most part (83.3%, supported by a positive DaTscan), could be categorized into mild early-stage PD.

The diagnosis of PD is largely based on the correct identification of its clinical features. In this regard, it is essential to differentiate early-stage PD from other forms of parkinsonism. To diagnose the early stages of PD, in addition to providing meticulous clinical examination of motor and non-motor clinical symptoms and signs, various other ancillary diagnostic tests such as DaTscan imaging, brain MRI, MMSE, and olfactory testing, and if necessary, MIBG myocardial scintigraphy and ^123^I-IMP SPECT, should be used, as elaborated in the following paragraphs.

First, dopamine transporter (DaT) imaging was the most indicative and predictive strategy to detect early PD in this study. Most patients (83.3%) with parkinsonian signs with a UPDRS-III score of 3 or 4 showed abnormal findings on DaTscan imaging. DaTscan can distinguish patients with PD from normal subjects even in early stages (10, 21), and also from patients with atypical parkinsonian syndromes (12) and non-degenerative forms of parkinsonism (21) such as essential tremor, vascular parkinsonism, and drug-induced parkinsonism. Furthermore, significant DaT changes may precede the onset of clinical symptoms (22). Among the motor symptoms of PD, tremor and rigidity do not correlate well with striatal DaT binding, whereas bradykinesia shows a significant correlation with DaT activity (23, 24). As regards 15 patients (16.7%) demonstrating no dopamine deficiency, some of them might be classified into a subgroup of clinically diagnosed PD patients having scans without evidence of dopaminergic deficit (SWEDD) (25), or their stages of parkinsonism might be too early to manifest as abnormal findings on DaTscan. Moreover, in this study, all the patients with subjective slowness of movement who underwent MIBG myocardial scintigraphy showed low cardiac MIBG uptake as well as a reduced SBR on DaTscan imaging, which is compatible with mild early PD. Although the alleged slowness of movement was not supported by objective evidence, it may imply a latent parkinsonian symptom.

Second, in the present study, olfaction in the patients with a UPDRS-III score of 3 or 4, including postural tremor with a positive DaTscan, was significantly impaired compared with that of controls, which shows that olfactory dysfunction may be one of the earliest clinical manifestations or preclinical symptoms of PD (26, 27).

Third, the current study revealed no reduced cognitive function as evaluated by the MMSE score or significant reduction of medial temporal structures using VSRAD software (18) in patients with subtle parkinsonian signs. Moreover, no significant reduction of blood flow was observed by ^123^I-IMP SPECT (3D-SSP) in patients with subjective cognitive decline. Regarding cognitive function, many reports refer to the association between cognitive impairment and parkinsonism (28, 29). Moreover, a higher prevalence rate of the coexistence of mild parkinsonism is reported in patients with MCI or mild AD (30). Therefore, parkinsonian signs recognized in this study should be substantially attributed to early PD rather than amnestic cognitive impairment.

The author admits the limitations of this survey: this is a non-multicenter study evaluated by one author, which may not vouch for the accuracy of UPDRS assessment; it is also a cross-sectional study; and the number of samples is rather small. However, it is important to shed a new light on subtle states between first symptoms and phenoconversion to manifest PD. In this context, the author believes that this study may help cover a gap between establishment of subtle parkinsonian signs and diagnosis of manifest PD. In order to replicate the findings in this study, more extensive epidemiological and clinical investigations in larger samples by multiple independent assessors would be needed, together with longitudinal data in the same patients. Such efforts would contribute toward uncovering the diverse spectrum of pre-diagnostic PD and, in turn, therapeutic strategies and prevention trials in PD.

## Data Availability Statement

The original contributions presented in the study are included in the article material, further inquiries can be directed to the corresponding author.

## Ethics Statement

The studies involving human participants were reviewed and approved by the ethics committee of Agano City Hospital. The patients/participants provided their written informed consent to participate in this study.

## Author Contributions

The author contributed to the conception and design of the study, the data acquisition, statistical analysis, execution of the research project, and wrote the manuscript.

## Conflict of Interest

The author declares that the research was conducted in the absence of any commercial or financial relationships that could be construed as a potential conflict of interest.
